# Endovascular management of post-pancreatectomy hemorrhage caused by a hepatic artery pseudoaneurysm: Case report and review of the literature

**DOI:** 10.1515/biol-2025-1127

**Published:** 2025-08-05

**Authors:** Ariadni Fouza, Ioakeim Giagtzidis, Maria Sidiropoulou, Elissavet Symeonidou, Anna Maria Kouskoumvekaki, Maria Daoudaki, Panagiotis Petras, Konstantinos Mpallas

**Affiliations:** 5th Surgical Department, Hippokratio General Hospital, School of Medicine, Aristotle University of Thessaloniki, Thessaloniki, 54642, Greece; Department of Radiology, Hippokratio General Hospital, School of Medicine, Aristotle University of Thessaloniki, Thessaloniki, 54642, Greece; Laboratory of Biological Chemistry, School of Medicine, Aristotle University of Thessaloniki, Thessaloniki, 54124, Greece

**Keywords:** hepatic artery pseudoaneurysm, post-pancreatectomy hemorrhage, endovascular management

## Abstract

Pancreaticoduodenectomy is the standard surgical treatment for a range of malignant and some benign diseases. The mortality rate associated with this procedure has decreased to less than 3% in recent years, although the morbidity remains high at 6–40%. Common complications may include delayed gastric emptying, pancreatic fistula, intra-abdominal abscess, and gastrointestinal or intra-abdominal bleeding, among others. Bleeding and pseudoaneurysm formation are likely to be the most significant complications. This is a case report about gastrointestinal bleeding following a Whipple procedure from an aberrant hepatic artery originating from the superior mesenteric artery (SMA), treated by endovascular means. The SMA was cannulated under local anesthesia and direct puncture of the common femoral artery. Catheterization and angiogram of the aberrant right hepatic artery identified the pseudoaneurysm and bleeding site at its bifurcation. Coil embolization resulted in pseudoaneurysm occlusion and bleeding management. Hepatic perfusion was not affected as the main vasculature of the liver, namely the common hepatic artery, remained intact. The management of hemorrhage following pancreatectomy represents a significant challenge, particularly given the vulnerability of the patient cohort and the necessity for re-operation in an anatomically challenging environment. Endovascular intervention is the preferred method of treatment when applicable, as it can be performed under local anesthesia and is associated with less morbidity.

## 1 Introduction

Pancreaticoduodenectomy (PD, also known as the Whipple procedure) is the standard surgical intervention for a range of malignant diseases, including carcinoma of the head of the pancreas, duodenal carcinoma, terminal bile duct malignancies, as well as certain benign conditions such as pancreatitis. The mortality rate of this procedure has decreased to less than 3% in recent years; however, the morbidity rate remains high, at 6–40% [1,2,3].

Complications may include delayed gastric emptying, pancreatic fistula, intra-abdominal abscess, and gastrointestinal or intra-abdominal bleeding [4,5]. Bleeding is considered to be the most serious complication, occurring in 6–10% of cases and accounting for 11–38% of all-cause mortality [4]. Pseudoaneurysms can be formed in 28% of those cases requiring urgent treatment [4].

This study is a case report of endovascular management of a hepatic artery pseudoaneurysm and gastrointestinal bleeding following Whipple’s procedure and literature review.

## 2 Case report

A 72-year-old male patient underwent a PD and right hemicolectomy for a pancreatic head carcinoma that had invaded the transverse mesocolon. On the 13th postoperative day, the patient experienced an episode of gastrointestinal bleeding, which was attributed to the ileo-transverse anastomosis. A reoperation was performed, during which the ileo-transverse anastomosis was resected, and a terminal ileostomy was created. Eight days following the second operation, the patient once again presented in the emergency department with acute gastrointestinal bleeding and hemorrhagic shock. A CT angiography (CTA) was conducted, which demonstrated the presence of hyperdense material within the gastrointestinal tract, suggestive of blood, yet no evidence of contrast extravasation was observed. The patient was treated conservatively with the transfusion of 11 units of packed red blood cells and 8 units of fresh frozen plasma.

**Figure 1:**
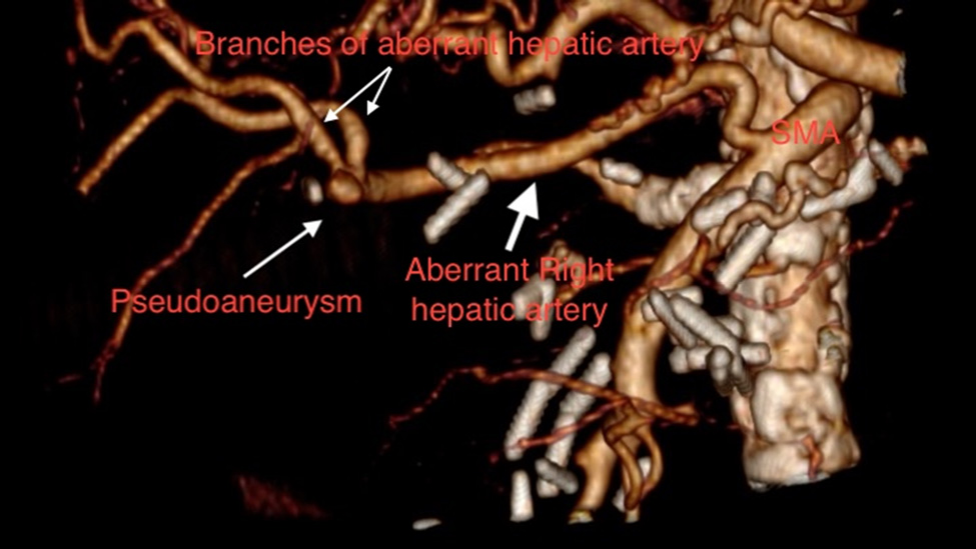
Anatomy explanation from the 3D reconstructed image.

**Figure 2:**
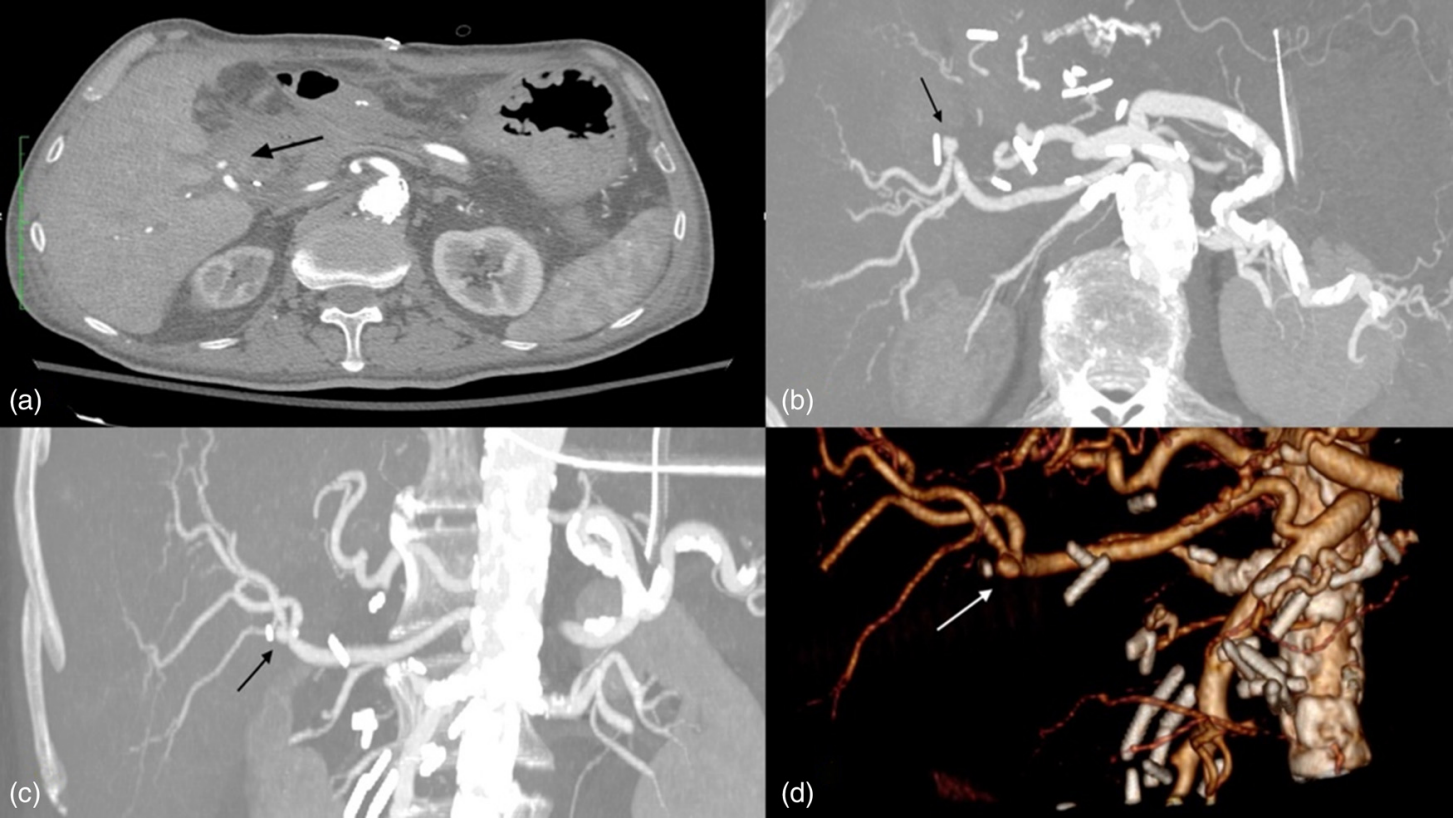
CTA. Arrows show hepatic artery pseudoaneurysm: (a) axial view, (b) axial MPR, (c) coronal MPR, (d) 3d reconstruction.

However, 2 days later, a more severe episode of gastrointestinal hemorrhage occurred. An urgent CTA was performed, which identified an aberrant right hepatic artery arising from the SMA and a pseudoaneurysm at its bifurcation (Figure 1) in close proximity to the hepaticojejunal anastomosis with a maximum diameter of 8 mm, without evidence of contrast extravasation (Figure 2).

**Figure 3:**
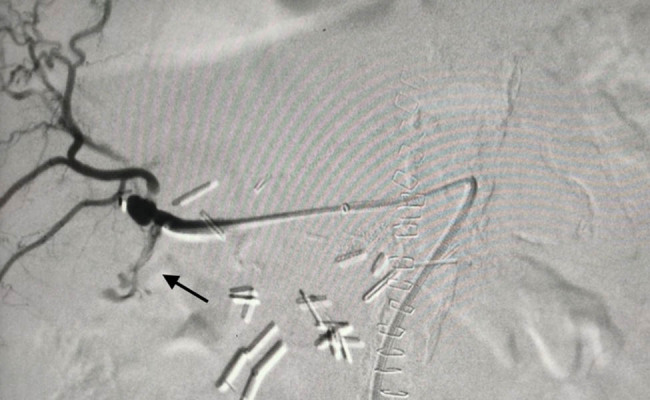
Intra-op angiography. The arrow shows the contrast extravagation.

**Figure 4:**
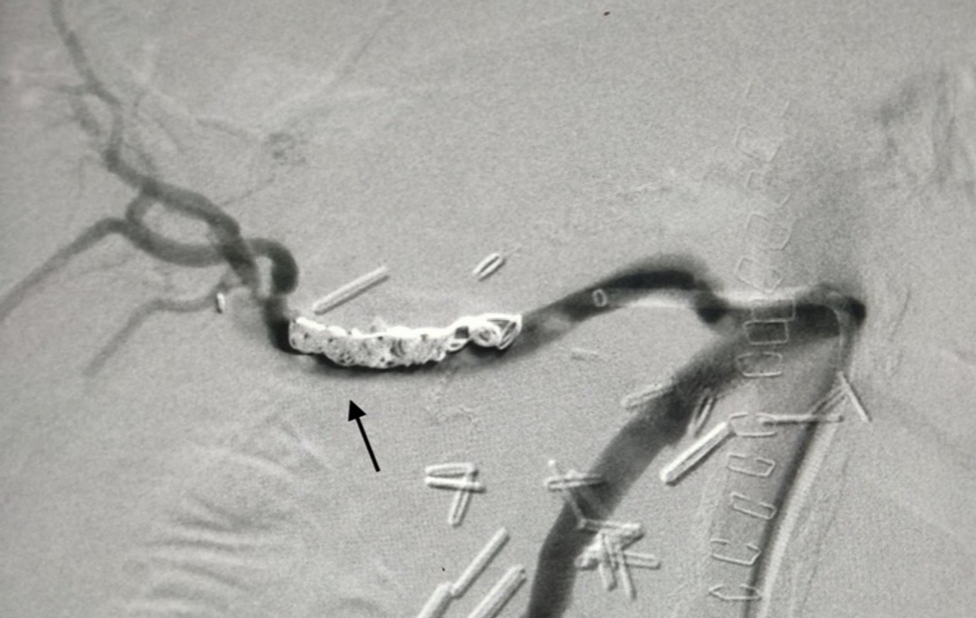
Final angiography with coils in place. No hemorrhage identified. Arrow indicates the previous site of extravagation.

An endovascular approach was deemed the optimal strategy. The SMA was cannulated with the use of a “Simmons” catheter under local anesthesia and direct puncture of the common femoral artery. A 6Fr-45cm sheath was advanced, and an angiogram revealed the aberrant right hepatic artery, as well as the pseudoaneurysm at the bifurcation of the hepatic artery (Figure 3). The original 0.035-in. stiff wire was replaced with a 0.035-in. “Rosen” wire, and a 5Fr “Vertebral” catheter was advanced distally beyond the pseudoaneurysm. In order to seal the pseudoaneurysm and the right hepatic artery, six detachable coils were deployed (Interlock-18 Fibered IDC occlusion system, Boston Scientific, Marlborough USA) of various sizes (8 × 100 *n* = 2, 6 × 100 *n* = 1, 4 × 100 *n* = 2, 4 × 60 *n* = 1). Stent graft deployment was not considered, since the location of the pseudoaneurysm at the bifurcation of the aberrant hepatic artery did not provide an adequate landing zone and diameter mismatch between the hepatic artery and its branches. Final angiography demonstrated successful occlusion of the pseudoaneurysm, without compromise of liver blood supply from the coeliac artery (Figure 4).

The postoperative period was uneventful, with no further instances of bleeding, and the patient was discharged on the fourth postoperative day. The patient’s hepatic function was not affected postoperatively, as the maximum values of hepatic enzymes were SGOT 58U/L and SGPT 68U/L. In fact, hepatic enzymes reached the highest measurement 2 days before the operation (SGOT 298U/L, SGPT 156U/L, ALP 1007U/L, γGT 320U/L). The deterioration of hepatic function preoperatively was attributed to ischemia due to hemorrhagic shock. The patient developed no further bleeding episodes 6 months postoperatively.


**Informed consent:** Informed consent has been obtained from the patient’s caregivers.
**Ethical approval:** The research related to human use has been complied with all the relevant national regulations, institutional policies and in accordance with the tenets of the Helsinki Declaration, and has been approved by the authors' institutional review board or equivalent committee.

## 3 Discussion

Post-pancreatectomy hemorrhage (PPH) is a common complication of the postoperative period in patients, with a mortality rate of 20–50% [6]. There is currently no consensus on the optimal management of PPH. A consensus statement on the definition and further categorization of the condition has been published by the International Study Group of Pancreatic Surgery (ISGPS) [4].

In accordance with the ISGPS classification system, PPH is initially categorized as either early or late. Early hemorrhage refers to incidences occurring within the first 24 h postoperatively and are typically attributable to erroneous surgical technique or underlying coagulopathies. The most common sites for bleeding are the retroperitoneum or omental branches, and such patients are predominantly managed with re-laparotomy [5,7]. Late hemorrhage occurs after the first 24 h and is associated with elevated mortality, particularly when it occurs after the fifth postoperative day.

PPH is additionally classified as either mild or severe. A mild hemorrhage is defined as a medium blood loss, with a drop in hemoglobin of less than 3 g/dL, hemodynamic stability, and recommended conservative treatment. Severe hemorrhage is characterized by a drop in hemoglobin of more than 3 g/dL, clinical signs of hemodynamic instability, and strong recommendation for active bleeding control. It is important to make a specific reference to the term “sentinel bleeding,” which refers to a limited amount of blood loss in the peritoneal cavity or the gastrointestinal tract. This type of bleeding is not clinically significant, it is characterized by a drop in hemoglobin of less than 1.5 g/dL, and it is typically resolved spontaneously [5].

Additionally, post-pancreatectomy bleeding may occur within the lumen or extraluminally. Intraluminal bleeding is evidenced by the presence of blood within the nasogastric tube, hematemesis or melena. During the initial three postoperative days, the source of bleeding is predominantly the gastroenteric or enteroenteric anastomoses, which are managed endoscopically with the use of epinephrine or clipping [1]. After the third day, intraluminal bleeding is derived primarily from the pancreaticojejunostomy and is initially managed with interventional angiography [8]. The term “false” extraluminal bleeding, as defined by Yekebas and Wolfram, describes intraluminal bleeding from the pancreaticoenteric anastomosis that results in its secondary disruption and subsequent bleeding into the peritoneal cavity [5]. Secondary leakage of pancreatic enzymes can result in vascular erosion and the formation of arterial pseudoaneurysms. It has been demonstrated that extensive dissection during lymphadenectomy can render vessels more vulnerable to pancreatic enzymes [9]. It is important to note that bleeding from pseudoaneurysms can manifest as intraluminal hemorrhage, whereby blood can enter the lumen of the bowel through a disruption in the pancreaticoenteric or hepaticoenteric anastomosis [30]. This was also the case in our patient [1].

Special reference should also be made to the anatomy of our patient, as the presence of the aberrant hepatic artery, which derived from the SMA allowed us to safely embolize this vessel, without completely disrupting the arterial blood supply of the liver, as the common hepatic artery (CHA) and its branches were left intact. Complete occlusion of the hepatic arterial blood supply could lead to biliary necrosis or hepatic abscesses. Miura et al. mention that the dual blood supply of the liver along with its extensive collateral pathways protect the organ from ischemic infarction and that occlusion of the hepatic artery is often tolerated. However, 5 out of 15 patients analyzed in their study suffered from infarction [6]. Yoon et al. revealed a 30% incidence of abscesses after hepatic artery occlusion [31], and Kim et al. found that six out of seven patients of their series developed hepatic abscesses [32]. Hassold et al. report a 29–70% liver ischemia in their study [13].

## 4 Endovascular management as first-line therapy

CTA is a mandatory procedure that plays an important role in the diagnosis and identification of PPH origin. The endovascular approach appears to be a promising and viable alternative to open repair, offering a minimally invasive procedure that can be conducted under local anesthesia [10,30].

Vascular access is typically obtained through the common femoral artery or the left brachial artery, and visceral angiography of the abdomen is conducted to identify instances of extravasation or the formation of pseudoaneurysms [11,12]. The most common sources of bleeding are branches of the SMA, the gastroduodenal artery stump, and the CHA and its branches [6]. The management of bleeding is achieved through embolization and/or the placement of a covered stent [11,13]. The choice of embolization agent is dependent on the specific circumstances. Coils, as well as other liquid embolization agents such as NBCA (*N*-butyl-2-cyanocrylate) glue, gelfoam, or zein ethiodized coil, may be utilized in this process [14,15,20]. There is no randomized prospective study or meta-analysis that compares different coiling embolization techniques and materials for arterial trauma or pseudoaneurysms. A comparative study from 2021 for partial splenic embolization came under the conclusion that permanent embolic materials achieved better laboratory and radiological outcomes than gelatin sponge particles in cirrhotic hypersplenism patients [33].

A literature review was conducted on a series of patients with PPH who had been treated by endovascular means using the PubMed database. The data collected included the location of the bleeding (intra or extraluminal), whether angiography was applied, and the responsible vessels (Table 1). Additionally, the use of embolization agents or stents, technical success, recurrent bleeding, and re-intervention, as well as overall mortality, were documented (Table 1). Case reports and patient series that did not include the aforementioned information were excluded.

**Table 1:** Literature review of studies with PPH

Author, year	Patient with PPH	Intraluminal	Extraluminal	Angio graphy	Source of bleeding	Pseudo aneurysm	Embolization/coil/stent (*n* = number)	Success
Yekebas et al. (2007) [5]	87	36	51	43 (49%)	HA (4)	4	Coil (*n* = 25)	20 (80 %)
					GDA/SPDA (12)			
					SMA branches (14)			
					SA branches (4)			
Xu et al. (2020) [10]	17	17	14	17 (100%)	GDA (15)	18	Coil (*n* = 18)	16 (94.1%)
					HA (1)			
					SA (1)			
Miura et al. (2007) [6]	15	NA	NA	12 (80%)	SMA (4)	5	Coil (*n* = 13)	6 (40 %)
					SMA + CHA (1)			
					HA (5)			
					GDA (6)			
Correa-Gallego et al. (2012) [7]	33	18	15	10 (30%)	HA (2)	2	Coil (*n* = 8)	8 (100%)
					GDA (4)			
					SA (1)			
					Pole branch of left renal artery (1)			
					Unknown (2)			
Khalsa et al. (2015) [1]	10	7	7	9 (90%)	GDA (4)	NA	Coil (*n* = 6), Stent (*n* = 3)	8 (89%)
					HA (2)			
					Jejunal branches of the SMA (1)			
					PDA (1)			
					Inferior phrenic (1)			
Izumi et al. (2023) [11]	6	2	4	6 (100%)	GDA (3)	3	Coil (*n* = 3), Stent (*n* = 3)	5 (83%)
					DPA (1)			
					PHA (1)			
					RHA (1)			
Pottier et al. (2015) [12]	69	9	60	69 (100%)	SA (14)	25	Coil (*n* = 47), Stent (*n* = 6)	51 (74%)
					GDA (13)			
					HA (8)			
					LGA (4)			
					Other (8)			
					No target artery (22)			
Hassold et al. (2016) [13]	27			27 (100%)	CHA (11)	6	Coil *n* = 11, Stent (*n* = 16)	24 (89%)
					GDA (5)			
					PHA (4)			
					SA (5)			
					SMA (3)			
					Right gastroepiploic (1)			
Ching et al. (2016) [14]	28	8	30		GDA (15)	26	Coil (*n* = 9), Stent (*n* = 25)	37 (97.4%)
					SMA (8)			
					HA (7)			
	38 Interventions-				SA (3)			
					PDA (3)			
					RHA (2)			
Asari et al. (2016) [15]	25	2	23	20 (80%)	CHA (5)	Unknown	Embolization (*n* = 25), Stent (*n* = 1)	16 (80%)
					PHA (3)			
					GDA (4)			
					SA (5)			
					SMA (4)			
					Unknown (5)			
					Other (11)			
Beyer et al. (2009) [16]	10			9 (90%)	GDA (5)	Unknown	Embolization (*n* = 8), Stent (*n* = 4)	9 (100%)
					Jejunal art. (2)			
					SA (1)			
					RGA (1)			
Darnis et al. (2013) [17]	46	26	20 (2 of parietal origin)	14 (30.4%)	SA (8)	Unknown	Embolization (*n* = 14)	7 (50%)
					GDA (3)			
					Other (4)			
					Unknown (3)			
Feng et al. (2014) [18]	73	40	45	19 (35.2%)	GDA (7)	9	Embolization (*n* = 11), Stent (*n* = 1)	9 (69.2%)
					PHA (4)			
					SMA (2)			
					CT (2)			
					CHA (1)			
					SA (1)			
					Other (2)			
Sanjay et al. (2012) [19]	11	2	9	8 (73%)	CHA (3)	6	Embolization (*n* = 5), stent (*n* = 3)	5 (62.5%)
					GDA (2)			
					SMA branches (2)			
					SA (1)			
Huo et al. (2015) [20]	21	15	11	18 (86%)	SMA (1)	Unknown	Embolization (*n* = 10), Stent (*n* = 8)	15 (71.4%)
					PHA (1)			
					GDA (11)			
					CHA (7)			
					IPDA (1)			
					SA (1)			
You et al. (2018) [21]	66	24	17	62 (94%)	GDA (26)	62	Embolization (*n* = 30), Stent (*n* = 19)	57 (92%)
					PHA (16)			
					CHA (14)			
					SMA (9)			
					Other (8)			
Choi et al. (2004) [22]	22	10	12	16 (73%)	GDA (5)	9	Embolization (*n* = 14)	8 (57%)
					CHA (3)			
					SMA (3)			
					PHA (1)			
					Other (2)			
Schäfer et al. (2010) [23]	18	7	4	12 (67%)	SA (4)	Unknown	Embolization (*n* = 12), Stent (*n* = 3)	10 (83%)
					SMA (1)			
					CHA (6)			
					RHA (3)			
					PHA (1)			
					GDA (2)			
					jejunal (1)			
Lee et al. (2010) [24]	27	10	13	23 (85%)	GDA (11)	23	Embolization (*n* = 18), Stent (*n* = 2)	18 (78.2%)
					HA (8)			
					Other (6)			
Makowiec et al. (2005) [25]	12	7	5	10 (83%)	CHA (5)	Unknown	Embolization (*n* = 4), Coil (*n* = 2)	6 (60%)
					GDA (5)			
					SA (1)			
					PDA (1)			
Ansari et al. (2016) [26]	19	12	11	10 (53%)	PHA (2)	Unknown	Embolization (*n* = 8), Coil (*n* = 2)	8 (80%)
					GDA (2)			
					RHA (1)			
					CHA (1)			
					Other (2)			
Zhang et al. (2011) [27]	14	4	10	11 (78.5%)	CHA (3)	Unknown	Embolization (*n* = 9)	12 (85.7%)
					RHA (1)			
					LHA (2)			
					CT (1)			
					SA (2)			
					GDA (4)			
					Unknown (1)			
Asai et al. (2014) [28]	32	28	18	32 (100%)	CHA/GDA (18)	Unknown	Embolization (*n* = 27), stent (*n* = 14)	25 (78%)
					SMA/IPDA (14)			
Stampfl et al. (2012) [29]	25	7	18	25 (100%)	IPDA (1)	8	Embolization (*n* = 23)	19 (83%)
					SMA branches (3)			
					GDA (7)			
					Other (3)			
					RHA (1)			
					PHA (4)			
					SA (4)			
					Unknown (2)			

CHA: common hepatic artery, CT: celiac trunk, DPA: dorsal pancreatic artery, GDA: gastroduodenal artery, HA: hepatic artery, IPDA: inferior pancreaticoduodenal artery, LGA: left gastric artery, LHA: left hepatic artery, PDA: pancreaticoduodenal artery, PHA: proper hepatic artery, RHA: right hepatic artery, SA: splenic artery, SMA: superior mesenteric artery, SPDA: superior pancreaticoduodenal artery. “Embolization” is referred to usage of coiling and other embolization agents.

The review included 24 studies conducted between 2007 and 2023 involving 713 patients with PPH. Angiography was performed in 510 cases (80.2%), resulting in 483 interventional procedures (67.7%) with a success rate of 75.7% (range 40–100%). The remaining procedures were conducted for diagnostic purposes only. The most common source of bleeding was the gastroduodenal artery (38.9%), followed by the hepatic artery and its branches (32.9%). The majority of PPH cases were managed with embolization (*n* = 370, 76.6%), while 125 (25.8%) patients had been treated with a covered stent, either as a primary intervention or in conjunction with embolization. The overall incidence of recurrent bleeding and the necessity for re-intervention was, on average, 19% (range 0–50%), while the mortality rate was 15.1%. A recent systematic review and meta-analysis on PPH came up with similar findings from our review [30]. Specifically, the success rate of an endovascular approach was 61%, with a mortality rate of 16%, while with an open surgical procedure, the success rate dropped to 56%, and the mortality rate increased to 37% [30].

It is evident from the existing literature that an endovascular approach is preferable in PPH cases [21,28] as it allows for minimally invasive treatment of frail and unstable patients with satisfactory technical success and outcomes. In the event of recurrent bleeding, which is a common occurrence, the benefit of embolization and/or stenting is that it can be performed again without the need for access to a hostile abdomen, which in turn lowers mortality rates. However, the surgical approach offers simultaneous control of septic sources, such as intraabdominal collections arising from pancreatic fistulas, and hemostasis with sutures allows control of arterial bleeding without completely blocking the vessels [6].

Endovascular techniques include embolization as well as covered stents, as mentioned above. Stents offer an advantage, as they maintain the blood flow of the vessels. However, it can be challenging to choose the correct size of the stent, as undersizing can lead to endoleaks, and oversizing can lead to vessel rupture [13].

## 5 Conclusion

The management of hemorrhage following pancreatectomy represents a significant challenge, particularly given the vulnerable nature of the patient cohort and the necessity for re-operation in an anatomically challenging environment. Advancements in CT technology can identify potential pathologies with great accuracy, while the broader accessibility of endovascular treatment has improved morbidity and mortality rates. Endovascular intervention appears to be the preferred method of treatment when applicable, as it can be performed under local anesthesia and is associated with less morbidity. This case report presents a “how to guide” for similar cases, with the use of available and low-cost materials, that can be performed in most vascular units with quite a steep learning curve.

A systematic review and a meta-analysis would clarify the best treatment option, the ideal embolization agent, and postoperative management for this complex and lethal complication.
